# Molecular and Cellular Advances in Endometriosis Research: Paving the Way for Future Directions

**DOI:** 10.3390/ijms241612663

**Published:** 2023-08-11

**Authors:** Antonio Simone Laganà, Federico Ferrari, Donatella Mangione, Fabio Fiorino, Alessandra Vassiliadis, Renato Venezia

**Affiliations:** 1 Department of Health Promotion, Mother and Child Care, Internal Medicine and Medical Specialties (PROMISE), University of Palermo, 90133 Palermo, Italy; donatella.mangione@unipa.it (D.M.); fabio.fiorino@unipa.it (F.F.); alessandra.vassiliadis@unipa.it (A.V.); renato.venezia@unipa.it (R.V.); 2 Department of Clinical and Experimental Sciences, University of Brescia, 25123 Brescia, Italy; federico.ferrari@unibs.it

Molecular and cellular research in the field of endometriosis is moving forward in giant steps. Indeed, the close collaboration among clinicians’ expertise in pharmacological and non-pharmacological approaches, minimally invasive surgeons, biologists with expertise in both basic and translational science, as well as biostatisticians for big data analysis are paving the way for future directions to be explored. The Special Issue “Molecular and Cellular Advances in Endometriosis Research 2.0” acted as a platform to collect some of the most interesting articles published recently in the field.

The molecular characterization of the differences between eutopic endometrium from women with endometriosis and ectopic implants is one of the main important challenges in endometriosis research [1], aiming to clarify the etiology of the disease [2]. In their work, Penariol and collogues emphasized ATF3, ID1, ID3, FOSB, SNAI1, NR4A1, EGR1, LAMC3, and ZFP36 genes and MT2A, TYMP, COL1A1, COL6A2, and NID2 proteins that were already reported in endometriosis, revealing integrated modulating signaling pathways such as the epithelial–mesenchymal transition (↑) and PI3K signaling via AKT to mTORC1 (↓ in proteome), mTORC1 signaling, TGF beta signaling, TNFA signaling via NFkB, IL6 STAT3 signaling, and responses to hypoxia via HIF1A targets (↑ in transcriptome) [3]. In addition, to differentiate between ectopic and eutopic endometrium, it is of paramount importance to obtain additional knowledge of the different types of endometriosis (peritoneal superficial endometriosis, ovarian endometriomas, and deep infiltrating endometriosis); in this context, an interesting study shed new lights on this topic, remarking how different phenotypes of endometriosis show an altered differential expression of genes and microRNAs in relation to adhesion and apoptosis pathways [4]. In addition, Istrate-Ofiţeru et al. found increased vascularity (CD34+) in areas with abdominal endometriosis and CD3+-:T-lymphocytes, CD20+-:B-lymphocytes, CD68+:macrophages, and Tryptase+: mastocytes were abundant, especially in cases with adenomyosis as a marker of the proinflammatory microenvironment, and a significantly higher division index-(Ki67+) in areas with adenomyosis, and the inactivation of tumor suppressor genes-p53+ in areas with neoplastic changes [5].

Indeed, accumulating evidence suggests that the local immune microenvironment may play a key role in the onset, development, and progression of endometriosis [6]. In this context, Krasnyi and coworkers found that the concentrations of ghrelin, GLP-1, glucagon, and visfatin in the peritoneal fluid were reduced in women with endometriosis [7]; at the same time, they noted an increase in CD10 protease expression by peritoneal macrophages, with a positive correlation of ghrelin and GLP-1 levels with CD86 macrophage expression in the study group; finally, a positive correlation was also found between the levels of GLP-1, glucagon, and visfatin with CD26 macrophage expression in peritoneal fluid, confirming the clear role of macrophages in the development of endometriosis [8]. From another perspective, Suszczyk et al. found an elevated percentage of myeloid dendritic cells and plasmacytoid dendritic cells with PD-L1 or PD-L2 expression and a higher concentration of the soluble forms of PD-L1 and PD-L2 in the peritoneal fluid than in the plasma of endometriosis patients [9], suggesting potential pathways to be elucidated for the onset of cancers from atypical endometriosis [10].

Overall, specific sex hormone polymorphisms were found to be associated with endometriosis risk and were involved in the molecular pathophysiology of the disease due to their functionality, as highlighted in the large genome-wide association study published by Golovchenko and colleagues [11], and this might explain, at least in part, the typical progesterone resistance associated with the disease, as summarized by Zhang and Wang [12].

Another main challenge in endometriosis research is the potential development of new strategies for the early and non-invasive diagnosis of the disease, as remarked by Ronsini et al. in their systematic review with high methodological quality [13]. Interestingly, Warzecha and collaborators found that the concentration of fibronectin in the plasma and peritoneal fluid in women with endometriosis was significantly higher than in the control group, regardless of the endometriosis stage, suggesting altered cell-extracellular matrix signaling [14].

These molecular and cellular advances might help to reduce the diagnostic delay associated with endometriosis, which has been further increased during the last few years to the COVID-19 pandemic [15], especially for the rare and non-typical presentation of the disease [16].

Considering the success and the high quality of the articles published in the Special Issue “Molecular and Cellular Advances in Endometriosis Research 2.0”, a new edition will be opened in the future aiming to collect the best research on the topic.
